# Endothelial Cords Promote Tumor Initial Growth prior to Vascular Function through a Paracrine Mechanism

**DOI:** 10.1038/srep19404

**Published:** 2016-01-14

**Authors:** Chengjian Zhao, Wei Zhang, Yuwei Zhao, Yun Yang, Hui Luo, Gaili Ji, E Dong, Hongxing Deng, Shuo Lin, Yuquan Wei, Hanshuo Yang

**Affiliations:** 1 State Key Laboratory of Biotherapy and Cancer Center, West China Hospital, Sichuan University, and Collaborative Innovation Center for Biotherapy, Chengdu, 610041, P. R. China; 2 Department of Molecular Cell and Developmental Biology, University of California Los Angeles, Los Angeles, California, USA

## Abstract

The angiogenic switch is an important oncogenic step that determines whether microtumors remain dormant or progresses further. It has been generally perceived that the primary function of this tumorgenic event is to supply oxygen and nutrients through blood circulation. Using *in vivo* imaging of zebrafish and mouse tumor models, we showed that endothelial cords aggressively penetrated into microtumors and remained non-circulatory for several days before undergoing vascular blood perfusion. Unexpectedly, we found that initial tumor growth in both models was significantly reduced if endothelial cords were removed by blocking VEGF-VEGFR2 signaling or using a vascular deficient zebrafish mutant. It was further shown that soluble factors including IL-8, secreted by endothelial cells (ECs) were responsible for stimulating tumor cells proliferation. These findings establish that tumor angiogenesis play a much earlier and broader role in promoting tumor growth, which is independent of vascular circulation. Understanding this novel mechanism of angiogenic tumor progression offers new entry points for cancer therapeutics.

Tumors originate as avascular multicellular aggregates and later induce angiogenesis when their sizes exceed a few millimeters due to hypoxia and nutrient deprivation^1^,^2^,^3^. The switch of tumors from an avascular to a vascular phenotype is called the ‘angiogenic switch’, which is critical for determining if a microtumor remains dormant or deemed to progress further^1^,^2^,^3^. Classic models of this angiogenesis imply that endothelial cells (ECs) infiltrate into microtumors as lumenized conduits^1^,^2^,^3^. However, how this process really occurs *in vivo* has not been clearly documented by high quality imaging studies.

Tumor blood vessels function to deliver oxygen and nutrients and remove waste products from tissues. Vascular circulation is a prerequisite for the proper function of vessels to sustain tumor growth^4^. *In vivo* studies of normal vascular development during embryogenesis have shown that blood vessels first form solid endothelial cords and subsequently lumenize to generate functional vessels permitting blood perfusion^4^,^5^,^6^,^7^,^8^,^9^,^10^,^11^. If a similar process takes place in microtumors during the angiogenic switch, there should be an initial stage in which a microtumor contains solid endothelial cords but no blood perfusion. This presumption triggered us to ask whether solid endothelial cords play any roles in regulating microtumor growth before the formation of functional vessels.

Vascular endothelium has been shown producing active substrates affecting normal development and function of several organs and tissues^12^,^13^,^14^. Studies have also demonstrated that angiocrine factors such as endothelin-1, basic FGF, TGF-beta, IL-6, and IL-8 positively impact on cancer progression^15^. In recent years, the paracrine effect of the vascular niche on modulating the homeostasis of tumor stem cells was further highlighted in different tumor types, including brain tumors and colorectal cancer^16^. Additionally, tumor associated ECs were found to function as a “chemo-resistant niche” or “radio-resistant niche” that promotes the survival and proliferation of residual tumor cells and serves as a reservoir for relapse^17^,^18^. Overall, findings from these studies offer conceptual basis for investigating roles of vascular ECs in supporting the growth and expansion of microtumors in a paracrine manner by angiocrine factors before the establishment of circulation.

To address this issue, we used both mouse and zebrafish tumor models coupled with imaging fluorescently labeled vascular ECs. Our studies indicate that the angiogenesis acts to promote microtumor growth by a two-phase model: endothelial cords in microtumors drive tumor growth through a paracrine mechanism by releasing endothelium-derived proliferative factors, then they support tumor progression by supplying oxygen and nutrients through the blood circulation.

## Results

### Endothelial Cords in Zebrafish Microtumor Xenografts

**Figure 1:**
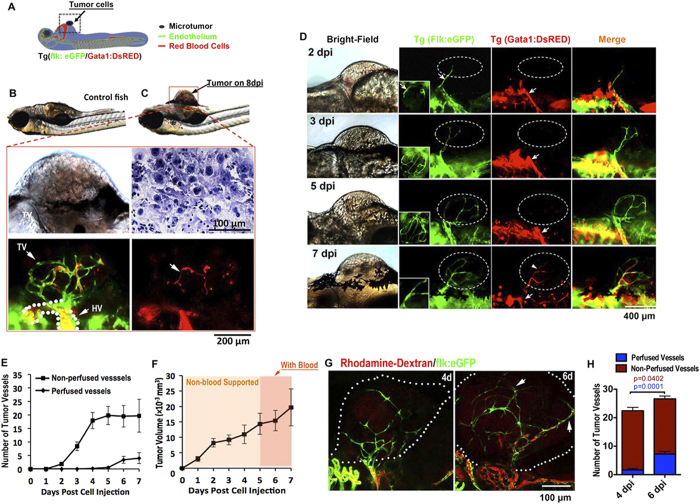
Solid endothelial cords penetrate into microtumor before blood perfusion in a zebrafish xenograft model. (**A,B**) Green tumor vessels and circulating red blood cells (red panel) in Tg(flk:eGFP; Gata1:dsRed) double transgenic zebrafish with mouse B16 melanoma xenografts. Hematoxylin staining shows the typical cytological features of tumor cells. TV, Tumor Vasculature. HV, Host Vessel. (**C,D**) Dynamic imaging of endothelial cords penetrate (green) into microtumors before blood perfusion (red). Blood flow (red) is observable in dilated host vessel, but absent in initial angiogenic sprouts (green) in microtumors (dotted circles) until 7dpi. Insets (**D**, green panels) indicate angiogenic sprouts from the ventral view. (**E,F**) Quantitative analysis of blood-perfused or non-perfused neovessels (**E**) and microtumor growth rate (**F**) in the xenografts (n > 20 for each day, error bars show SEM). (**G**) Microangiograph by injecting Rhodamine-Dextran (red, 2 million MW) from posterior cardinal vein showing the absence of blood low (red) in solid endothelial cords (green) in 4dpi xenografted microtumors (dotted circles), but present on 6dpi. (**H**) Quantitative analysis indicates the change of tumor neovessels with or without blood-perfusion on 4dpi and 6dpi (n = 15 for 6dpi, n = 12 for 4dpi, error bars show SEM).

To simultaneously investigate the infiltration of angiogenic sprouts into microtumors and the emergence of blood circulation in microtumors (Diameter <1 mm) *in vivo*, we established a xenograft tumor model in Tg(flk1:eGFP; Gata1:dsRed) double transgenic zebrafish (Fig. 1A)^19^,^20^. In this model, the dynamics of neovascularization in microtumors, including the growth of angiogenic sprouts and the establishment of blood perfusion can be imaged under fluorescent microscope at high-resolution (Fig. 1B,C)^19^,^20^,^21^,^22^. Neoangiogenic sprouts were first observed to project from host vessels into xenografts (mouse B16 melanoma) on 1 day post implantation (dpi) (500–1000 cells per embryo) (Fig. 1D and Supplementary Fig. S1). The number and length of endothelial sprouts increased until day 4 or 5, but blood perfusion within tumors was rarely observed (Fig. 1E,F). Microangiogaphy by injecting Rhodamine-dextran into the circulation of tumor-bearing Tg(flk:eGFP) zebrafish embryos showed that non-circulatory endothelial sprouts occupied the microtumor and lacking observable blood circulation on 4dpi, but blood perfusion generally happened after 6 or 7 dpi (Fig. 1G,H). Even until 8dpi, solid endothelial cords remain coexisted with hollow vascular vessels within tumor xenografts (Supplemental Fig. S2). Thus, these *in vivo* tracking results from the xenografted mouse melanoma in zebrafish established that “angiogenic switch” consisted of a fairly long period of solid endothelial cord stage induced by microtumors.

To confirm that this phenomenon was not limited to mouse melanoma, mouse CT26 colon cancer xenografts were implanted in zebrafish embryos. Again, abundant numbers of solid endothelial cord were consistently observed around 5dpi (Supplementary Fig. S3A,B). Furthermore, considering immortal cell lines may exhibit altered properties due to prolonged culture *in vitro*, we prepared and cultured human glioblastoma (GBM) cells^23^. By implanting multicellular spheres of cultured GBM into the transgenic zebrafish, formation of solid endothelial cord was observed between 9 and 15 dpi (Supplementary Fig. S3C).

### Endothelial Cords in Endogenous Zebrafish Microtumors

**Figure 2:**
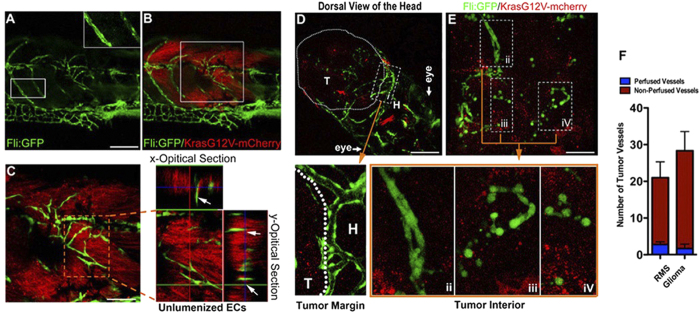
Solid endothelial cords are recruited into the endogenous microtumors in zebrafish. (**A,B**) Hollow host vessels (inset box) at the margin of endogenous rhabdomyosarcoma (RMS) and solid endothelial cords (green) at the interior of RMS microtumor (red), in a 25d Tg(fli1a1:GFP) zebrafish. (**C**) 3D projection and optical section images of the interior RMS microtumor (box in **C**) showing the tumor-recruited solid angiogenic sprouts. (**D,E**) Endogenous glioma and blood vessels in the head of 9d zebrafish. Hollow host vessels (panel i) in head (**H**) is morphologically distinct to solid endothelial sprouts or separate ECs in glioma (panels ii, iii, iv). (**F**) Quantitative analysis of tumor neovessels with or without blood-perfusion in RMS and glioma (n = 3 for RMS, n = 4 for glioma, error bars show SEM). For experimental outline and the resulting RMS and glioma bearing zebrafish, see Fig. S4.

The findings described above could be due to some undefined incompatibilities between the two different species. To rule out this possibility two zebrafish endogenous tumors models were established and inspected. Rhabdomyosarcoma (RMS) and Glioma were induced by co-injecting plasmids of UAS-mCherry-KrasG12V and Rag2-Gal4VP16 or GFAP-Gal4VP16 to Tg(fli1a:eGFP):p53 +/− transgenic zebrafish embryos at the one cell stage as previously described^24^ (Supplementary Fig. S4). Generally, externally visible glioma developed as early as 8 days post fertilization (dpf) in zebrafish, while RMS could not be detected until 20 dpf. Three-dimensional projection and sectioning of the 25 dpf RMS showed the high-density endothelial cords in the Kras-mCherry + microtumor, compared with the neighboring well-organized and hollow host vessels (Fig. 2A–C). High-magnification double fluorescent images of both the interior and marginal areas of Glioma at 9 dpf in the zebrafish brain clearly showed the hollow host vessels at tumor margin (Fig. 2D, *i*) and solid endothelial cords or scattered cells in the interior microtumor (Fig. 2E, ii–iv). Confocal images of the 9 day glioma (n = 3, Diameter <1 mm) and 25 day RMS (n = 4, Diameter <1 mm) showed most of the tumor associated endothelial cells at the early stage of tumor progression were arranged as solid endothelial cords without perfusion in the microtumors (Fig. 2F). These results suggested that solid endothelial cords within microtumors prior to the blood-perfusion are a general phenomenon both for xenograft and endogenous tumors in zebrafish.

### Endothelial Cords in Mammalian Tumor Models

**Figure 3:**
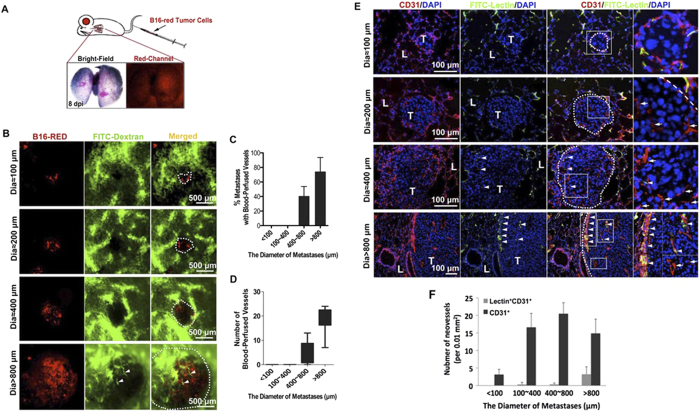
Endothelial cells infiltrate into pulmonary metastases before the blood perfusion. (**A**) The pulmonary metastatic model in mice was established by inoculating red fluorescence-labeled B16 melanoma cells into the tail vein (shown as the cartoon drawn by Zhao C.J.). A representative image of 8d mouse lung with red-fluorescent (red channel) black (Bright-Field) melanoma metastases is shown below. (**B**) Micrometastases (red) in mouse lungs and vessels with active circulation (green, 70 k MW FITC-dextran, arrowheads) were detected under stereomicroscope. Dotted lines show the location of metastases. (**C,D**) Quantification of blood-perfusion in micrometastases of different diameters, and the number of blood-perfused vessels in micrometastases of indicated diameter range (80 micrometastases for each group, error bars show SEM). (**E**) Endothelial cells within blood-perfused (CD31^+^ Lectin^+^, arrowheads) or non-perfused (CD31^+^ Lectin^-^, arrows) vessels were detected in frozen sections. L, lung tissue; T, tumor. The areas in the white boxes are magnified further in the far right panels of each row. (**F**) Quantitative analysis of the number of CD31^+^ endothelial sprouts and Lectin^+^ CD31^+^ blood-perfused neovessels (>20 selected areas in each group are counted, error bars show SEM).

To further confirm the finding of the aggressive penetration of solid endothelial cords before blood-perfusion in microtumor is not limited to the zebrafish, we tested it in a mouse tumor model. A pulmonary metastatic model in mice was established by inoculating red fluorescence-labeled B16 melanoma cells into the tail vein and metastases in the lungs could be detected under stereomicroscope 8 days later (Fig. 3A and Supplementary Fig. S5A–C). Two kinds of FITC-dextran fluorescent dyes (70k and 200k MW) were injected intravenously to visualize blood-perfused vessels. In this setting, tumor vessels with active blood circulation can be easily identified by their bright green fluorescence. Avascular microtumors were pure red colonies (Fig. 3B and Supplementary Fig. S6). Mice were sacrificed on day 8 after tumor cell inoculation; microtumors ranging from 50 μm to 1000 μm in diameter were checked under a stereo fluorescence microscope. Tumors (red) with diameters >800 μm had significantly more blood-perfused vessels (bright green) than tumors ranging from 400 to 600 μm in diameter. However, no circulatory vessels were observed in microtumors (pure red) that were less than 400 μm in diameter (Fig. 3B–D and Supplementary S6A).

Next, to test the existence of ECs within the non-perfused microtumors (Diameter <400 μm), we further labeled blood-perfused and non-perfused vascular vessels in these microtumors separately and simultaneously^25^. FITC-lectin was intravenously injected to label the internal ECs of vessels with active blood circulation, and CD31 immunostaining was performed on the same lung samples to detect all the ECs in microtumors^25^. Similar to FITC-dextran results, this study revealed that CD31^+^ Lectin^+^ ECs (ECs belong to blood-perfused vessels) were present in the marginal regions of relatively bigger microtumors (diameters >800 μm), were occasionally observed in 400–600 μm microtumors, and were absent in diameter <400 μm ones (Fig. 3E,F). However, CD31^+^ Lectin^-^ ECs (ECs belong to non-perfused vessels) were present in microtumors of almost all sizes, including those less than 400 μm, whereas CD31^+^ Lectin^+^ blood-perfused ECs were restricted to the marginal regions of larger tumors (Fig. 3E,F). Similarly, when the same experiment was performed using CT26 colon cancer cells, penetration of non-circulatory ECs into the microtumors (Diameter <400 μm) was also observed (Supplementary Fig. S5D,E). To further confirm the fluorescent staining results, we examined the mouse pulmonary microtumors using electron microscope and separate ECs were observed locating in the central regions of mirotumors (Diameter <400 μm), while hollow tumor vessels were only present in bigger pulmonary tumors (Diameter >1 mm) (Supplementary Fig. S7).

Together, observations in mice and zebrafish propose a unique phase during the initial vascularization for microtumor, in which angiogenic sprouts exist as solid endothelial cords before they transform into hollow vascular vessels permitting blood perfusion into microtumors.

### Endothelial Cords are Required for Tumor Initial Growth in Zebrafish

Given that solid endothelial cords that did not permit blood circulation but were associated with an obvious initial growth phase of microtumors (Fig. 1), we hypothesized that they might stimulate microtumor expansion independent of blood-perfusion.

**Figure 4:**
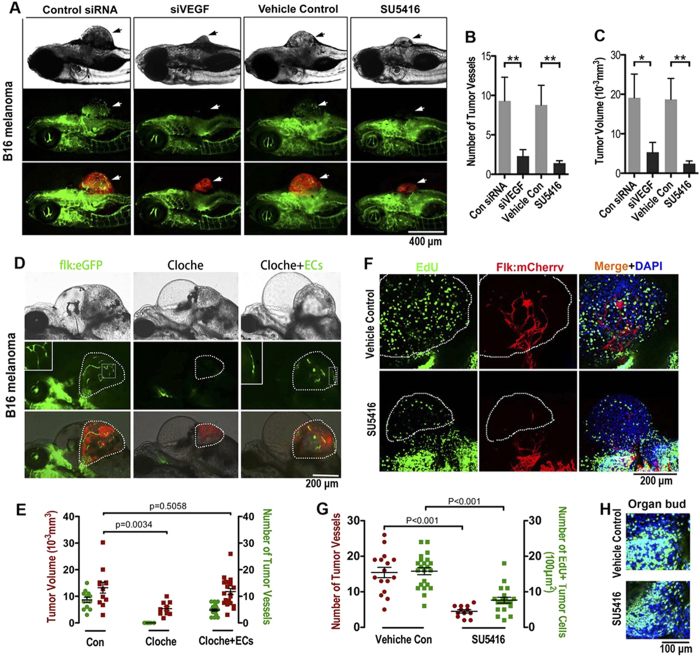
Endothelial cords are required for initial tumor growth in zebrafish. (**A**) Simultaneous dual fluorescence imaging showing the xenografted mouse melanoma (red) and endothelial cords (green panel, arrows) in 5d Tg(flk:eGFP) transgenic zebrafish using tumor cells transfected with control siRNA or VEGF siRNA, or using zebrafish recipients treated with SU5416 (2 μM) or control vehicle. (**B,C**) Quantitative analysis of tumor-induced endothelial sprouts and the tumor volume at the day 5 (n > 50 fish for each group; *p < 0.05, **p < 0.01, error bars show SEM). (**D**) Slower growth of xenografted microtumor (red) in zebrafish *cloche* mutant is significantly rescued by exogenous ECs (green) sorted from 24 hpf Tg(flk:eGFP) zebrafish. Dotted lines indicate the location of microtumor. (**E**) Quantitative analysis of tumor-associated endothelial cells and the xenografted microtumor volume (n > 11 fish for each group, error bars show SEM). (**F**) Number of proliferating tumor cells (EdU+, green) in microtumor in Tg(flk:mCherry) transgenic zebrafish decreased when solid endothelial cords (red) are blocked by SU5416 (2 μM) treatment. Dotted lines indicate the location of microtumors. (**G**) Quantitative analysis of tumor-associated endothelial cells and the EdU + proliferating tumor cells density (n > 12 fish for each group, error bars show SEM). (**H**) Cell proliferation in normal developing organ buds is not affected by SU5416 treatment at the same dosage.

To block the initial penetration of solid endothelial cords into the microtumors, B16-Red cells were transfected with VEGF siRNA prior to transplantation or blocked VEGFR2 by using selective small molecule inhibitor SU5416. Experiments were terminated at 5 dpi when blood has not perfused into microtumors (Fig. 1D). Both treatments significantly blocked the penetration of endothelial cords into microtumors. Interestingly, initial tumor growth before the emergence of blood perfusion was dramatically reduced (Fig. 4A–C). Importantly, both VEGF siRNA transfection and SU5416 (1 and 2 μM) treatment did not show cytotoxic effects on tumor cell proliferation *in vitro* (Supplemental Fig. S8A–D).

To further confirm that the elimination of solid endothelial cords by either siVEGF or SU5416 treatment was the basis for the interruption of initial microtumor growth, B16 tumor cells were transplanted into the Tg(flk1:eGFP) transgenic zebrafish embryos carrying the *Cloche* mutation, which specifically lacks ECs and blood cells^26^. We found that the growth of xenografted microtumor in *cloche* mutant was significantly slower than that on the control fish (Fig. 4D,E). However, when we mixed ECs sorted from wild type Tg(flk1:eGFP) embryos (24hpf) with the transplanted tumor cells (ratio:1 to 10), initial growth of microtumor in *cloche* mutant was partially rescued (Fig. 4D,E). Similar to those endothelial cords penetrating into microtumors in Tg(flk1:eGFP), exogenous ECs in microtumors in flk1:eGFP/cloche fish were also able to undergo morphological changes into cord-like phenotype (Fig. 4D, cloche+ECs panel). Because blood circulation was completely absent in cloche mutant (Fig. 4D, cloche panels)^26^, we concluded that endothelial cords or ECs play a unique role of stimulating the initial microtumor growth independent of blood circulation.

To determine whether these initially penetrated endothelial cords or ECs stimulated tumor cell proliferation, EdU (100 μM) was added into the transplantation buffer before tumor cells injection, and the incorporation of EdU in proliferating tumor cells were analyzed at 5dpi. The results showed that much more EdU+ proliferating cells in control tumors than those in smaller tumors lacking endothelial cords or ECs in by SU5416 treated zebrafish (Fig. 4F,G). Notably, this dose of SU5416 treatment has no observable affects to cell proliferation in normal tissue (Fig. 4H). Thus, endothelial cords or ECs in the microtumor promots tumor growth by stimulating tumor cell proliferation.

### Endothelial Cords are required for Tumor Initial Growth in Mice

**Figure 5:**
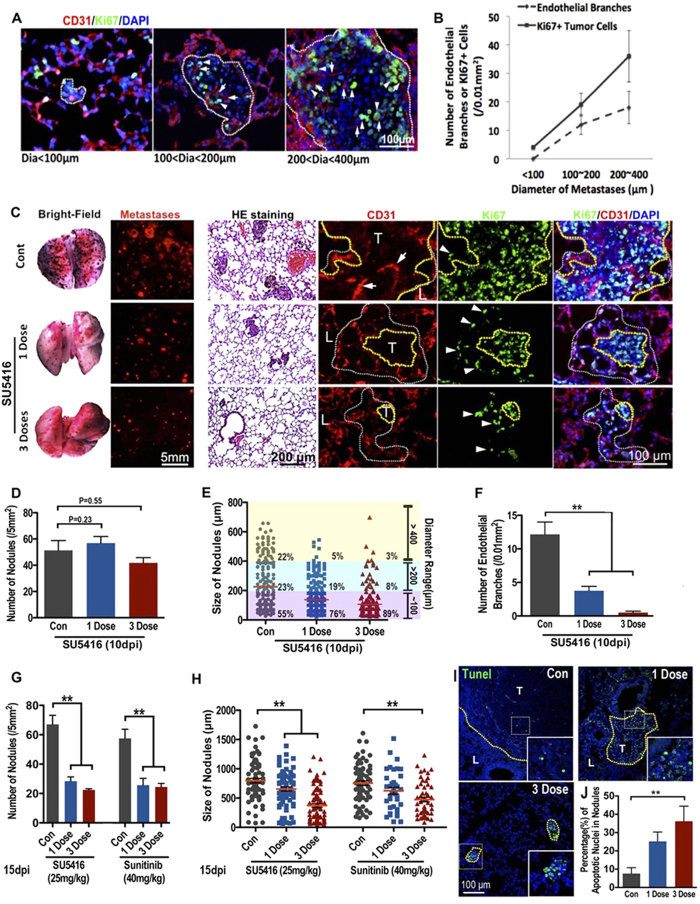
Endothelial cords are required for the expansion of pulmonary metastases in mouse. Double immunofluorescence staining (**A**) and quantitative analysis (**B**) of proliferating tumor cells (Ki67^+^, arrowheads) and tumor-induced endothelial cells (CD31, arrows) in pulmonary metastases with different diameters (n > 20 metastases for each group are counted, error bars show SEM). (**C**) SU5416 treatment impaired both ECs penetration and initial pulmonary metastases expansion. Images show 10 dpi mouse lungs with B16 metastases (black spots in bright-field or red spots in fluorescent panel. H&E staining shows the pulmonary metastases in mice lungs, CD31 and Ki67 double immunofluorescence staining shows the ECs (arrows) and proliferating tumor cells (arrowheads). Yellow dotted line is based on DAPI staining to indicate the outline of the main tumor clones. White dotted line is based on Ki67 and DAPI staining to indicate the range of proliferating tumor cells. (**D–F**) Quantitative analysis of the tumor-induced ECs, size and density of pulmonary metastases of 10d mouse lungs treated with or without SU5416. (**G,H**) Quantitative analysis of the density and size of pulmonary metastases under stereomicroscope of 15d mouse lungs treated with either SU5416 or Sunitinib. (n > 20 selected areas in each group, n = 5 individual mice lung are counted).( *p < 0.05, **p < 0.01, error bars show SEM). (**I,J**) Representative image and quantitative analysis of apoptosis in pulmonary metastases in mice treated by SU5416. **p < 0.01.

To determine whether endothelial cords or ECs play a role in mouse microtumor progression, we first investigated the association of proliferating tumor cells to tumor ECs in different sizes of B16 mouse pulmonary metastases (n = 20 for each size subgroup). Double immunostaining results showed that within microtumors without blood perfusion (<400 μm, Fig. 3) the Ki67+ cells in microtumors were proximal to CD31+ endothelial cells (Fig. 5A), and more ECs and Ki67+ cells were detected in bigger microtumors (Fig. 5B), suggesting ECs may play an important function during initial tumor growth prior to blood perfusion.

To investigate whether solid endothelial cords is critical to the initial growth of microtumor *in vivo*, we first determined the chronological emergence of angiogenic sprouts and blood perfusion in growing metastases in mouse lungs. By respectively tracking the growth of microtumors and blood perfusion at 3, 5, 7 and 9 days post tumor cell seeding, we found blood perfusion into the microtumors in the mouse lung mainly started on day 7 (tumor diameter = 553 ± 89 μm, dotted blue line; 10 of 92 had blood-perfused vessels, Supplemental Fig. S9A–C). SU5416 was then administered intraperitoneally on day 6 (1 dose) or day 5, 6, 7 (3 doses) post B16-red cell seeding to inhibit the penetration of solid endothelial cords or ECs into the mouse pulmonary metastases before blood perfusion. Mice were sacrificed on day 10 or day 15. Significantly bigger melanoma microtumors were found on the control lungs compared with the SU5416 groups at 10 dpi (Fig. 5C). Double immunostaining of CD31 and Ki67 showed that SU5416 treatment caused a dose-dependent inhibition of CD31^+^ECs into microtumors. Meanwhile, a marked decrease in the tumor mass size and Ki67^+^cells number were also observed. Moreover, we noticed that Ki67^+^ tumor cells in ECs-deficient microtumors that treated by SU5416 were more widely scattered, and all scattered Ki67^+^ tumor cells were closely associated with neighboring CD31^+^ECs (Fig. 5C). Although there was a big difference of the size of metastases between the control lungs and SU5416 groups on 10dpi (Fig. 5C–F), we were still able to observe similar counts of total microtumors between the control and SU5416 groups using the more sensitive florescence microscope (Fig. 5C,D). This finding suggests that the lack of ECs dramatically impaired the expansion of microtumors. After 5 days of further growth, substantial differences were observed not only in the size but also in the number of microtumors between the SU5416 and the control groups at 15 dpi (Fig. 5G,H and supplemental S10). TUNEL staining indicated a higher apoptosis ratio of tumor cells in metastases of SU5416 treated lungs, further suggested that the penetration of ECs or endothelial cords before blood perfusion is necessary for the initiation growth of pulmonary metastases (Fig. 5I,J). To further test our results, we tried a FDA approved RTK inhibitor-Sunitinib, which is also a strong VEGFRs inhibitor besides its effects on tumor proliferation. We found, similarly to the SU5416 results, a single dose (administered on day 6) of Sunitinib (40 mg/kg) could significantly inhibit the expansion of B16 pulmonary metastases (Fig. 5G,H).

### Endothelial Cell-Derived Anigocrine Factors stimulating Proliferation of Tumor Cells

**Figure 6:**
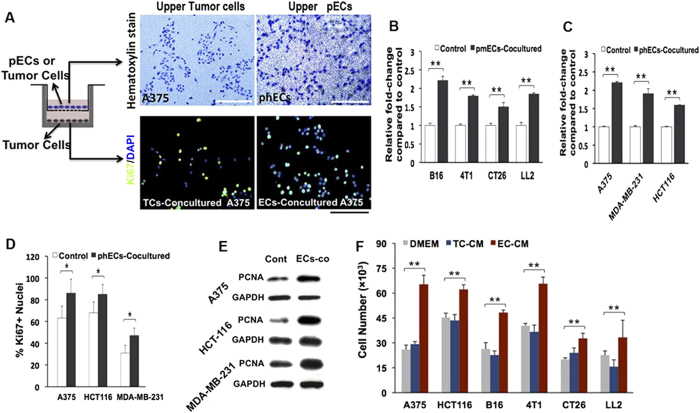
Endothelial cell-derived anigocrine factors stimulate the proliferation of tumor cells. (**A**) Diagram showing the co-culturing system with tumor cells seeded in the base wells and primary mouse and human endothelial cells (pmECs and phECs) or control cells (syngeneic tumor cells) in the upper chambers; Hematoxylin staining of co-cultured tumor or endothelial cells in the upper chambers (top panel) and Ki67 immunofluorescence staining of tumor cells in the base wells (bottom panel). (**B,C**) Quantitative analysis of the tumor cells in the base wells after 4 days’ coculturing. Mouse B16 melanoma, 4T1 breast, CT26 colon, and LL2 lung cancer cells are co-cultured with pmECs; Human A375 melanoma, MDA-231 breast and HCT116 colon cancer cells are co-cultured with phECs. (**D**) Quantitative analysis of the Ki-67 + tumor cells in base wells (n > 20, selected areas are counted for each group). (**E**) Western-blot showing the expression of PCNA in base wells. (**F**) Quantitative analysis of the function of EC-CM (endothelial cells conditioned medium) to the growth of tumor cells. TCM (tumor cell conditioned medium) and DMEM media are used as control (n = 5 wells for group). (*p < 0.05, **p < 0.01, error bars show SEM).

Given that solid endothelial cords cannot deliver oxygen and nutrients, we hypothesized that “angiogenic factors”^27^ secreted by ECs may be responsible for stimulating tumor cell proliferation before blood perfusion. To test this, we isolated primary mouse ECs (pmECs) and primary human ECs (phECs) and co-cultured them with different mouse or human tumor cells, in a trans-well system that only allows the exchange o tors between chambers^28^. After co-culturing in serum-reduced medium for 4 days (medium was changed once after 48 h), tumor clones in the lower base wells were counted and examined by Ki67 immunostaining and proliferating cell nuclear antigen (PNCA) Western blot, and co-cultured pECs or control cells in the upper wells were stained by Hematoxylin. Results showed that larger tumor clones were observed in the base wells that co-cultured with pECs (Fig. 6A–C). Also, we found tumor cells in the base wells were associated with a higher percentage of Ki67+ cells and an increased PCNA expression (Fig. 6D,E). Further, EC-Conditioned Medium (EC-CM) was collected from 48 h tumor cells and ECs co-culturing system and used to treat corresponding tumor cell lines. After 4 days of EC-CM treatment (medium changed every other day), increased growth rate of different tumor cell lines were observed, comparing with that treated by regular medium and pure tumor cell conditioned medium (Fig. 6F). These results together suggested that ECs promote tumor cell proliferation in a paracrine manner by secreting soluble angiocrine factors.

### Endothelial Cell-derived IL8 stimulating tumor growth *in vitro* and *in vivo*

**Figure 7:**
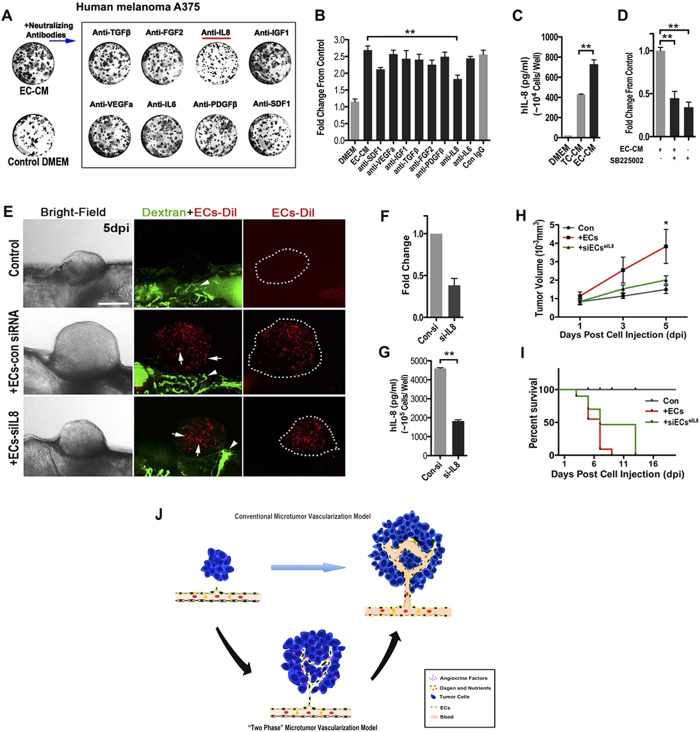
ECs-derived IL-8 mediated the initial growth of A375 micro-xenografts *in vitro* and *in vivo*. (**A**) Representative images of human A375 melanoma clones cultured in phEC-CM supplemented with different neutralizing antibodies for 4 days. (**B**) Quantitative analysis of the A375 tumor cells in phEC-CM with different neutralizing antibodies. (n = 5 wells for each group). (**C**) Quantitative analysis of the A375 tumor cells in phEC-CM treated with SB225002 (1 μM, inhibitor of IL-8 receptor CXCR2) inhibitor (n = 5 wells for each group). (**D**) hIL-8 concentration of DMEM, TC-CM and EC-CM (n = 3 wells for each group). (**E**) Representative images of A375 xenografts with or without incorparated ECs (Arrows), primary ECs (HUVECs) were pre-stained by CM-Dil, blood flow (Arrowheads) in zebrafish were imaged by FITC-dextran (2 million MW). Dotted lines indicate the location of microtumors. (**F,G**) Evaluation of IL-8 expression by q-PCR and Elisa at 24 h after ECs was treated by siRNA. (**H,I**) Quantitative analysis of xenografts growth rate and the survive rate of zebrafish hosts after implanted with A375 tumor cells alone or hECs: A375 cells mixture (1:10). (n > 20 fish for each group). (**J**) The “two-phase” model of ‘angiogenic switch’. Angiogenic neovessels are conventionally believed infiltrating into the avascular microtumor as circulatory sprouts with blood perfusion. We propose that initial endothelial cords in microtumors remain non-circulatory and drive tumor growth through a paracrine mechanism by releasing endothelium-derived proliferative factors (angiocrine factors), before they support tumor progression by supplying oxygen and nutrients through the blood circulation.

To identify the potential factors that contribute to promote tumor cell proliferation in EC conditional medium (EC-CM), we used a panel of neutralizing antibodies to specifically block some of endothelial cell-derived soluble factors that were previously reported^27^. Antibodies against human VEGF, IGF-1, FGF2, PDGF, TGF-β, IL-6, IL-8 and SDF-1 were individually added to primary human ECs conditional medium (phEC-CM) before treating tumor cells. Blocking IL-8 significantly impaired the proliferative effect of phEC-CM on human A375 melanoma cells (Fig. 7A–C), consisting with the inhibition of IL-8 receptors CXCR1 and CXCR2 by using specific small molecular inhibitor SB225002 (Fig. 7D and supplemental Fig. S11). In addition, immunodepletion of IL-8 from phEC-CM also caused a significant reduction of cancer cell growth, while the add-back of exogenous IL-8 partially but significantly rescued the effect of IL-8 depletion (Supplemental Fig. S12). These results suggested the functional importance of ECs-derived IL8 for the initial growth of A375 microtumors. To further confirm this *in vivo*, we detected the effects of IL8-deficient ECs to A375 xenografts growth on zebrafish. When A375 cells were implanted into zebrafish embryos, the xenografts tend to be dormant or grow very slowly. However, if A375 cells were pre-mixed (10:1) with primary human ECs before injection, the growth of A375 xenografts was dramatically accelerated (Fig. 7E–H) and most of tumor recipients died within 10 days (Fig. 7I). However, the pre-silence of IL8 in phECs by IL8 siRNA before tumor implantation could significantly slow down the growth of A375 xenografts (Fig. 7E–H) and delay the death of recipients from tumor progression (Fig. 7I). To confirm whether IL-8 also overexpresses in human primary melanoma ECs, we double stained the expression of CD31 and IL-8 in human melanoma tissues and found human melanoma ECs indeed had a high expression of IL8 (Supplemental Fig. S13).

Interestingly, when we repeated our neutralizing antibody blocking experiment using other tumor cell lines, including human HCT116 colon cancer cells, mouse B16 melanoma cells and mouse CT26 colon cancer cells, different angiocrine factor combinations were identified (summarized in Fig. S14A). However, IL-8 and FGF2 are the most common factors identified as proliferating stimulators in EC-CM. In zebrafish, we isolated ECs (GFP-positive, 2.2% of whole cell population) from Tg(flk:egfp) zebrafish endogenous glioma before active blood circulation occurs and detected the expression of IL-6, IL-8, FDF2 and SDF-1 by using quantitative RT-PCR. The result showed that FGF2 has the highest about six times higher expression in EC cords-derived cells than in non-EC cells (Supplemental Fig. S14B).

Collectively, we proposed a ‘two-phase’ model that solid endothelial cords firstly penetrated and played a role in stimulating initial tumor cells proliferation in a paracrine manner by releasing endothelium-derived soluble factors (‘angiogenic factor’, e.g. IL-8 and FGF2), followed by lumenization allowing the blood perfusion to supply oxygen and nutrients to support the further tumor growth (Fig. 7J).

## Discussion

Tumor vasculature is essential for tumor initiation, progression and metastasis. The proper function of tumor-associated vasculature was originally thought only to depend on blood circulation in the continuous vascular lumen. Studies have shown that the establishment of functional vascular vessels is a complex multi-step process. In normal embryonic development, ECs that migrate into the interstitial spaces first have to form an unlumenized cord (non-circulatory) followed by subsequent lumenization to generate a functional vessel through several distinct hollowing mechanisms^4^,^5^,^6^,^7^,^8^,^9^,^10^,^11^. No definite *in vivo* evidence exists to demonstrate whether this “two-stage” process is true during tumor neovascularization. In the current study we clearly show that tumor angiogenesis also occurs by the “two-stage” model. Namely, the ECs that initially infiltrate into tumor tissue assemble into solid endothelial cords and then become circulatory with obvious lumens permitting blood circulation. We confirmed these observations in both in zebrafish and mouse tumor models, establishing that the “two-stage” process of tumor angiogenesis is a general phenomenon.

According to the prevailing view to tumor vasculature, solid endothelial cords should be non-functional in promoting tumor growth until they become circulatory conduits. Here we found that disrupting the establishment of solid endothelial cords resulted in severe impairment for the initial tumor progression both in mouse and zebrafish models. The results indicate that unlumenized blood vessels are not merely static structural constituents of blood vessels, but also functional in regulating tumor growth in a blood perfusion-independent manner. It is well known that tumor vascular structures are highly disorganized and a great number of endothelial cell networks in tumors are not connected to circulation. Therefore, it is possible that those no-blood-perfused vessels in macrotumor tissues may be undergoing the first phase of an angiogenic process and are actively promoting tumor progression in a circulation independent manner. This might explain why the whole tumor microvessels density (MVD) has a close relationship to tumor progression and patient prognosis, despite of the fact that only part of these tumor blood vessels are lumenized and blood-perfused. The endothelial cell constitutes the main building block of tumor vessels^4^,^10^. Our findings are consistent with several reports that ECs themselves are not solely passive conduits for delivering oxygen or nutrients, but have direct functions in a paracrine manner in physiological and pathological conditions. For example, ECs support the normal development of pancreas and liver^12^,^13^,^29^, the self-renewal and repopulation of hematopoietic stem cells^30^,^31^ and neural stem cells^28^, the regeneration of liver and lung^32^,^33^ and growth of human colorectal cancer cells^34^.

We demonstrated that ECs promote proliferation of tumor cells through releasing a combination of selective angiogenic factors, such as FGF2, IGF-1, IL-8, IL-6, and SDF1. In support of this result, it has been shown that tumor cell-derived VEGFA can activate ECs to release multiple trophogens to support the expansion of leukemic cells, including VEGF, IL-1, IL-6 and nitric oxide (NO)^27^,^35^,^36^. Also, ECs are shown to interact closely with self-renewing brain and colorectal cancer cells by secreting factors that maintain these cells in a stem cell-like state^16^,^34^. We propose that the endothelial cell is a novel type of stromal cell that can directly drive initial tumor growth, in addition to known cell type such as fibroblast stromal cells^37^.

We envision that disrupting the formation of non-lumenized angiogenic circuits by targeting ECs in mictotumor or micrometastases represents a promising approach to prevent tumor recurrence and metastasis. Based on the prevailing concept of tumor angiogenesis that tumor growth is dependent on the capacity to acquire oxygen and nutrients through the blood supply, current anti-angiogenic therapeutic approaches are designed to disrupt this process to “starve” tumor cells by inhibiting the functional sprouting and assembly of abnormal tumor vessels using anti-angiogenic agents (i.e. anti-VEGFA Bevacizumab)^38^. However, emerging evidence suggests that anti-angiogenesis treatment paradoxically results in many undesired consequences^39^. Anti-angiogenesis induced hypoxia influences many crucial aspects of cancer biology and is strongly associated with the malignant alteration of the entire tumor and resistance to therapy including tumor angiogenesis, vasculogenesis, and the epithelial-to-mesenchymal transition^39^,^40^,^41^. Therefore, despite the efficacy of some antiangiogenic agents in improving the survival of tumor-bearing mice, so far the clinical application of antiangiogenic agents only shows a transient benefit to patients. Most patients ultimately surrender to tumor progression^42^. We suggest an alternative approach; in which endothelial derived pro-tumorigenic growth factors (angiocrine factors) but not existing tumor vessels are targeted. The function of blood vessels – delivering oxygen and nutrients as passive conduits – would not be disrupted. Therefore, the anti-angiocrine factor treatment would not encourage tumor hypoxia and would not lead to subsequent malignant alterations that usually caused by traditional anti-angiogenesis therapy, e.g. rebounding tumor angiogenesis, local invasiveness and distal metastasis, chemoresistance and radioresistance. Also treatment with anti-angiocrine factors would not result in the decrease of drug distribution in tumor tissue when combined with other therapeutic approaches such as chemotherapy. Therefore, this novel anti-tumor approach would provide better clinical benefits than traditional anti-angiogenic strategies by combining currently available anti-tumor applications, not only for cancer treatment, but also for preventing recurrence and metastasis. Importantly, it should be noted that ECs in individual organs are endowed with organ-specific phenotypic and functional attributes^3^,^27^,^32^,^33^,^43^, and the panels of soluble angiogenic factors released would differ among individual ‘tumor societies’. Consequently, individually identifying tumor-specific angiocrine factors is a prerequisite for developing effective anti-angiocrine factor therapeutic treatments.

## Experimental Procedures

For complete experimental details, see Supplementary Information

### Cell and Cell Culture

Mouse and human tumor cells were cultured as previously described^44^. Primary human ECs (phECs) were isolated from the human umbilical cord vein^45^. Primary mouse pulmonary ECs (pmECs) were isolated from 4–6-week-old wild-type C57BL/6 mice lungs^46^. A detailed primary cell isolation and culturing method is described in the Supplemental Experimental Procedures.

### Mice and zebrafish

Female C57BL/6 or BALB/c mice, 4–8-week old, were cared and used in standard facilities in Sichuan University. Zebrafish were bred and maintained normally (temperature, 28 uC; pH 7.2–7.4; 14 hr on and 10 hr off light cycle). All animal experiments were performed according to the guidelines of the Animal Care and Use Committee of Sichuan University (Chengdu, Sichuan, China) and approved by the institutional review board of the Medical Faculty at the West China Hospital, Sichuan University.

### Mouse pulmonary metastatic model

A suspension of 2.5 × 10^5^ red fluorescently labeled B16-red cells or CT26 cells in 0.1 ml of cell culture medium was injected into mouse through the tail vein. Mice were sacrificed at different time points and mouse lungs were excised and treated if needed. See the Supplemental Experimental Procedures for details.

### Xenograft Model in Zebrafish

Tg(flk1:eGFP), Tg(flk1:mCherry), Tg(Gata1:DsRed), Tg(fli1a:GFP), Tg(lysozymec:GFP) and Tg(MPO:GFP) transgenic zebrafish and zebrafish carrying P53 mutant and Cloche mutant were used in this project. All the fish used were bred and maintained normally (temperature, 28 °C; pH 7.2–7.4; 14 hr on and 10 hr off light cycle). 5 ~ 10 nanoliters suspension containing about 50–100 mouse B16 cells or 500 human A375 cells were implanted into 48hpf (hours post fertilization) zebrafish embryo through the perivitelline space in a single injection by using an electronically regulated air-pressure microinjector (Harvard Apparatus, NY, PL1-90). See the Supplemental Experimental Procedures for details.

### Endogenous tumor models in zebrafish

Zebrafish Rhabdomyosarcoma (RMS) was induced by co-injecting of Rag2-Gal4VP16 and UAS-mCherry-KrasG12V into zebrafish embryos at the one cell stage at a final combined concentration of 50 ng/μl as described^47^. Similarly, zebrafish spontaneous Glioma was induced by co-injecting of GFAP-Gal4VP16 and UAS-mCherry-KrasG12V into zebrafish embryos at one cell stage at a final combined concentration of 20 ng/μl.

### Imaging and neovessel quantification

Digital micrographs were taken with a Zeiss Imager.Z1 fluorescence microscope (Carl Zeiss Microimaging Inc., Germany) or a Zeiss LSM 510 Meta Confocal Microscope (Carl Zeiss Microimaging Inc., Germany). The tumor size, vessel length and vessel diameter were based on fluorescent images and quantified by Axiovision Rel 4.8 software (Carl Zeiss Microimaging Inc., Germany). See the Supplemental Experimental Procedures for details.

### EdU cell proliferation assay

Detection for the proliferating tumor cells in the zebrafish was done using Click-iT® EdU Alexa Fluor® 488 Imaging Kit (Invitrogen, C10337). The experiments were done mainly following the manufacturer’s instructions with some modification. See the Supplemental Experimental Procedures for details.

### Transwell Coculture experiments

For the coculture experiments, 10,000 tumor cells were seeded into the 24-well plates, and 10,000 corresponding tumor cells or primary ECs were seeded into upper compartments (transwell inserts, pore size: 0.4 μm, sigma-Corning), serum reduced DMEM (2% FBS) was added into the base wells (600 μL) and the upper transwell inserts (200 μL). See the Supplemental Experimental Procedures for details.

### Immunofluorescence

Frozen tissue was cut into 8-μm-thick cross-sections for the double immunofluorescent staining. The primary antibodies were rat anti-CD31 (BD PharMingen, 561814), and rabbit anti-Ki67 (ABCAM, ab15580). Host specific Alexa Fluor 488– and 594–conjugated secondary antibodies (Invitrogen) were used to reveal the primary antibodies. The nuclei were counterstained with 4′-6-Diamidino-2-phenylindole (DAPI, Sigma). See the Supplemental Experimental Procedures for details.

### Histology Analysis

Frozen xenografts on zebrafish embryos and B16 metastases in mouse lungs were sectioned at 8 μm thickness, and H&E staining was performed as standard protocol. See the Supplemental Experimental Procedures for details.

### Cytokine Neutralizing Assay

To neutralize cytokines in HUVEC (phECs) conditioned media, antibodies against human TGFβ (Abcam, ab27969), FGF2 (Millipore, 05-117), IL8 (Abcam, ab10769), IL6 (Abcam, ab6672), IGF1 (Abcam, ab9572), VEGFa (Millipore, 07-1420), PDGFβ (Abcam, ab9704) and SDF1 (Abcam, ab10395) were used. To neutralize cytokines in pmECs conditioned media, antibodies against mouse TGFβ (Abcam, ab64715), FGF2 (Abcam, ab33103), VEGFa (R&D, AF-493-NA), IL6 (R&D, AF-406-NA), IL-1a (R&D, AB-400-NA), and IGF1 (R&D, AF791) were used. See the Supplemental Experimental Procedures for details.

### Statistical Analysis

Data was assayed by unpaired student’s t test using Prism statistical analysis software. A level of *P < 0.05 or **P < 0.01 was regarded as statistically significant.

## Additional Information

**How to cite this article**: Zhao, C. *et al.* Endothelial Cords Promote Tumor Initial Growth prior to Vascular Function through a Paracrine Mechanism. *Sci. Rep.* **6**, 19404; doi: 10.1038/srep19404 (2016).

## Supplementary Material

Supplementary Information
