# Supplementary material for: Endothelial Cords Promote Tumor Initial Growth prior to Vascular Function through a Paracrine Mechanism
Source: Sci Rep. 2016 Jan 14;6:19404. doi: 10.1038/srep19404 (PMC4725875; doi:10.1038/srep19404)
Supplement: Supplementary Information [file srep19404-s1.pdf]

Endothelial Cords Promote Tumor Initial Growth prior to Vascular Function through a Paracrine Mechanism

Chengjian Zhao[1,△], Wei Zhang[1,△], Yuwei Zhao[1], YunYang[1], Hui Luo[1], Gaili Ji[1], Er Dong[1], Hongxing Deng[1], Shuo Lin[1,2], Yuquan Wei[1], Hanshuo Yang[1,*]

1. State Key Laboratory of Biotherapy and Cancer Center, West China Hospital, Sichuan University, and Collaborative Innovation Center for Biotherapy, Chengdu, 610041,P. R. China.
2. Department of Molecular Cell and Developmental Biology, University of California Los Angeles, Los Angeles, California, U.S.A.

Supplemental Figures and Table

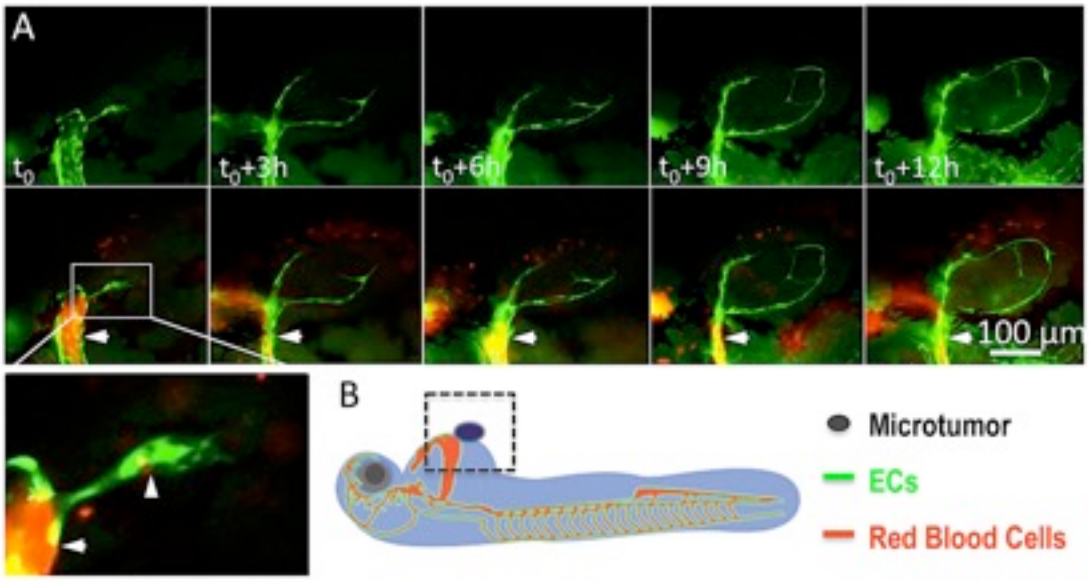

Figure S1. Penetration of endothelial cords into xenografted microtumor on Tg(flk:eGFP::Gata1:dsRED) double transgenic zebrafish. (A) Real-time observation showing tumor-induced endothelial sprouts (panel t_0, arrowhead in magnified image) from the dilated host vessel (arrow in A) on 1dpi are solid and tend to attract each other as they stretching in the microtumor mass. (B) Diagram shows the strategy of establishing the xenograft tumor model by injecting 500-1000 tumor cells besides 48hpf zebrafish common cardinal vein (CCV).

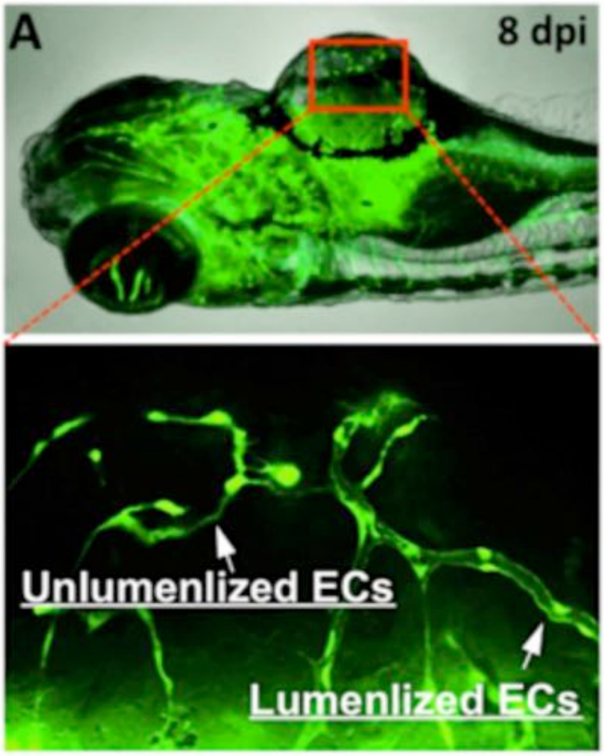

Figure S2. Lumenization of solid angiogenic sprouts in xenografted microtumor on zebrafish. (A) The coexistence of lumenized and unlumenized angiogenic circuits in xenografted tumors at 8 dpi (days post cell injection). (B) The formation of endothelial lumens via the dynamic fusion of intracellular vacuoles (arrows). (C) Schematic shows the process of endothelial cord lumenization in the xenografted tumor in zebrafish.

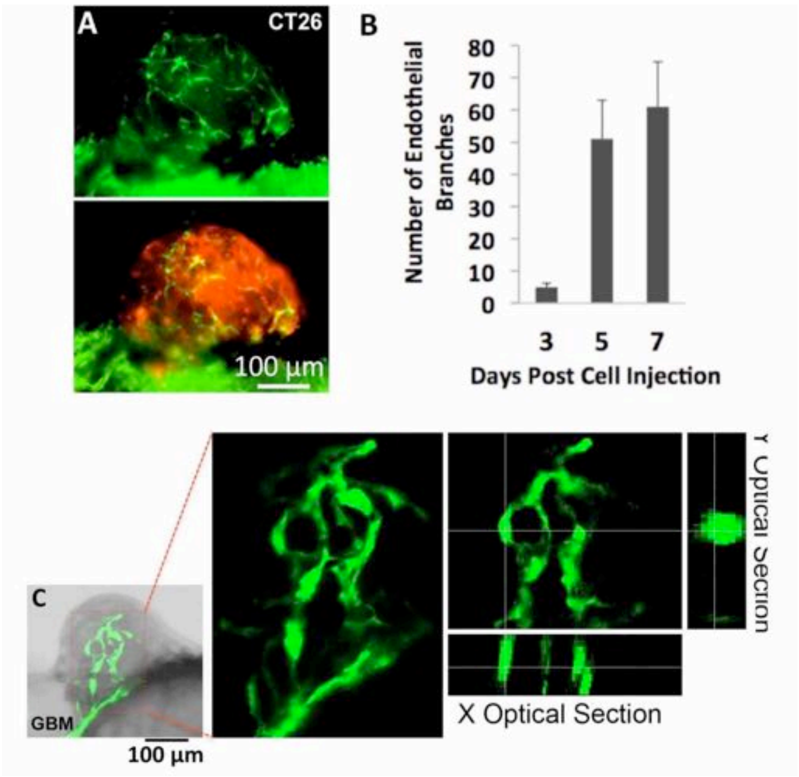

Figure S3. Endothelial cords in xenografted CT26 microtumors and human glioblastoma (GBM) in zebrafish. (A) A CT26 microtumor (red, stained by FAST Dil) with endothelial cords in Tg(flk: eGFP) zebrafish on 6 dpi. (B) Quantitative analysis of the number of endothelial branches in the microtumors with time (>20 for each group, scale bars show SEM). (C) Endothelial cords in human GBM xenograft in zebrafish.

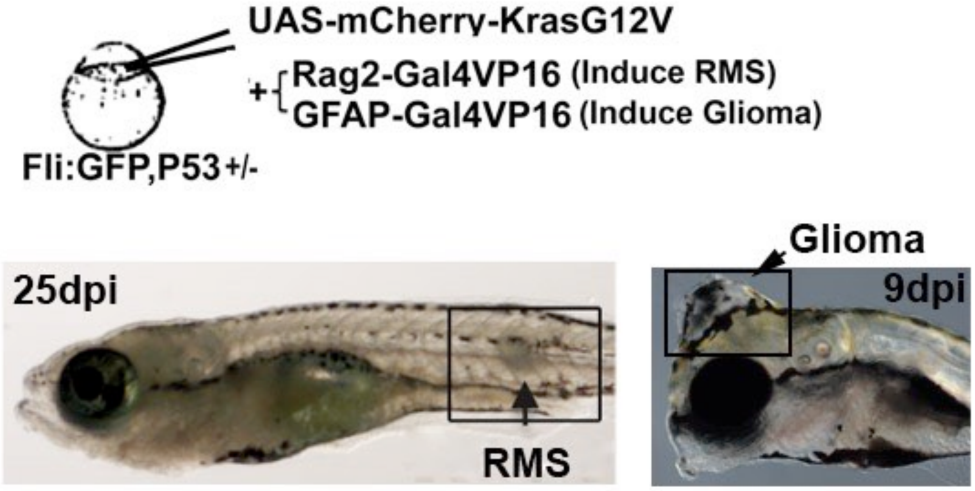

Figure S4. The strategy for inducing endogenous tumors in Tg(fli:GFP):P53+/- transgenic zebrafish by co-injection of UAS-mCherry-KrasG12V with Rag2-Gal4VP16 for Rhabdomyosarcoma (25dpi), or GFAP-Gal4VP16 for Glioma (9dpi). Phenotypes are shown for each tumor type.

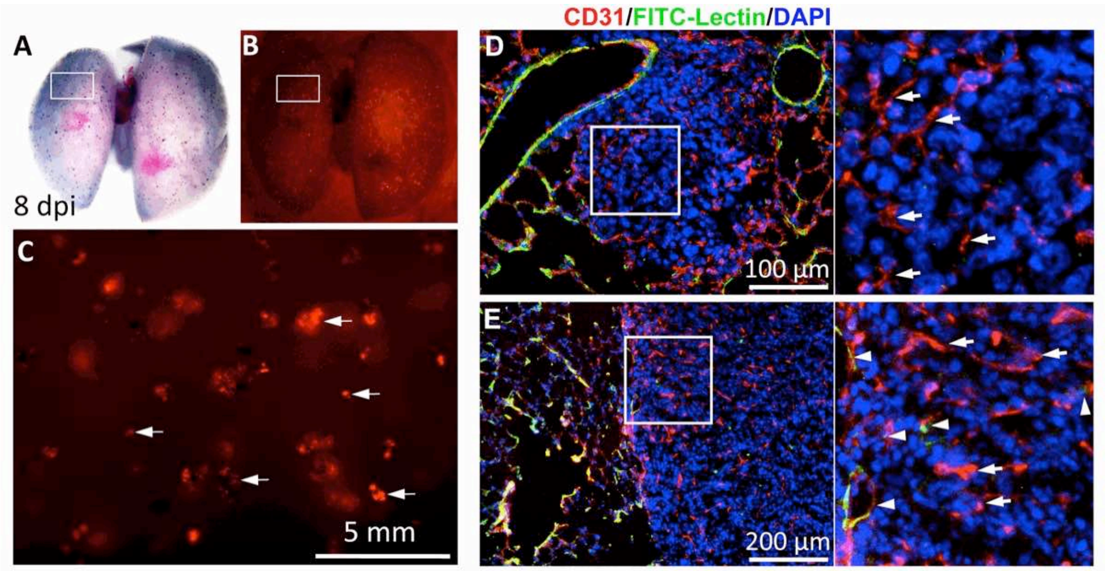

Figure S5. The pulmonary metastatic model in mice. (A-C) The mouse lung with B16 micrometastases on 8 days post cell injection. Arrows indicate micrometastases. (D, E) Non-perfused blood vessels ($CD31^+Lectin^-$, arrows) in Dia<400μm CT26 colon cancer micrometastases (D) and coexist with blood perfused vessels ($CD31^+Lectin^+$, arrowheads) in Dia>800μm micrometastases (E) in mouse lungs. Right panels show magnified views of the indicated areas (white box).

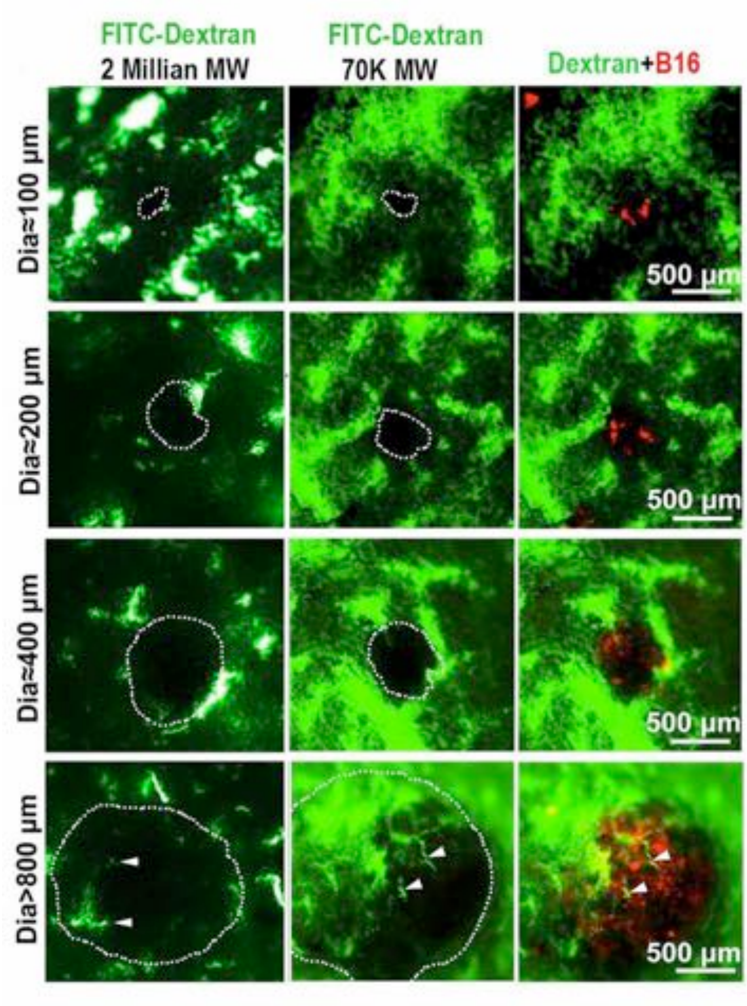

Figure S6. (A) Blood-perfusion (FITC-dextran) in the B16 pulmonary metastases is not visible until a tumor size reaches 400 μm. Melanoma pulmonary metastases (black clones with red fluorescence), blood perfusion (70k and 2 Million MW FITC-dextran, arrowheads). Dotted line separates the host lung tissue from the tumor.

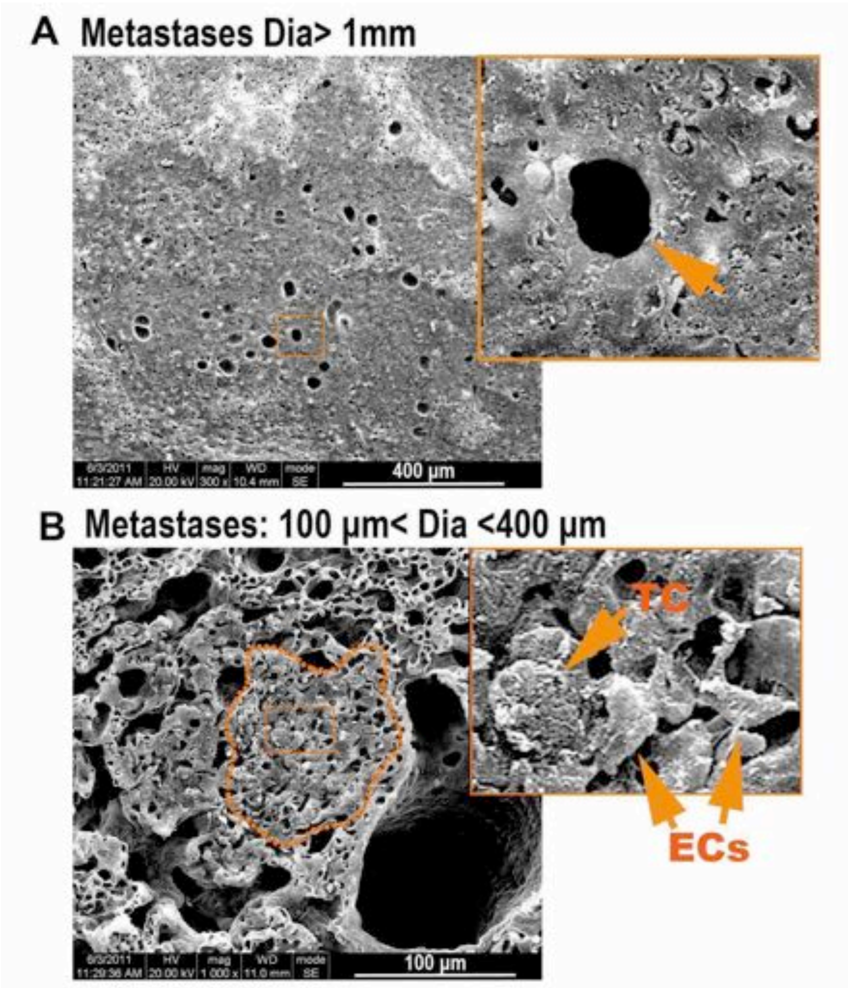

Figure S7. Scanning electron microscope image of mouse B16 pulmonary metastases. (A) Pulmonary B16 micrometastases with diameter >1mm, tumor vessel (arrow). (B) Pulmonary B16 micrometastases with diameter ranging from 100 μm to 400 μm, tumor cell (TC, arrowhead), Endothelial cells (ECs, arrows); dotted line separate micro tumor from normal tissue; areas in boxes were magnified right.

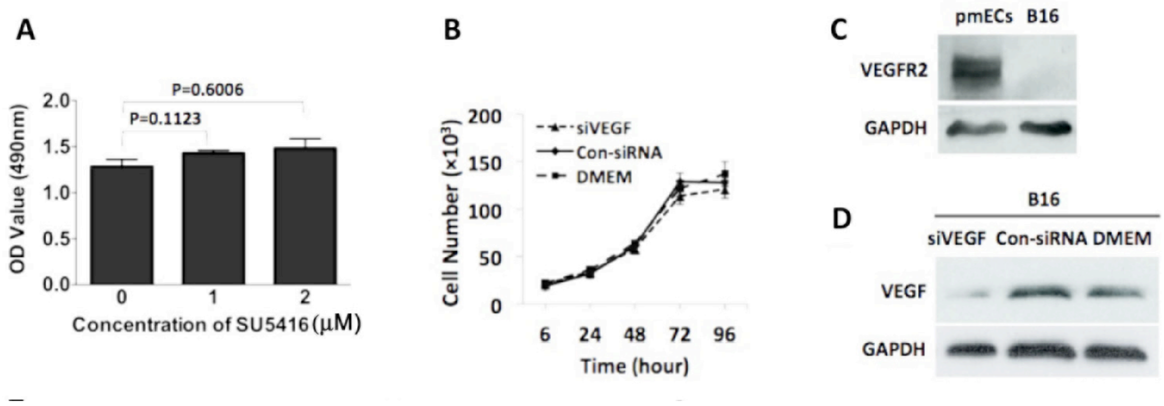

Figure S8. Inhibition of VEGF/VEGFR2 signal pathway has no effect on the proliferation of B16 cells *in vitro*. (A) The growth of B16 tumor cells treated by SU5416 was measured by MTT assay, p>0.1 by t test, 5 wells for each group. (B) The number of tumor cells at different time points after siRNA transfection was counted under microscopy after trypsinization. (C) Expression of VEGFR2 in primary ECs, and B16 cells. (D) Knockdown of VEGF expression in B16 cells by siRNA-VEGF transfection.

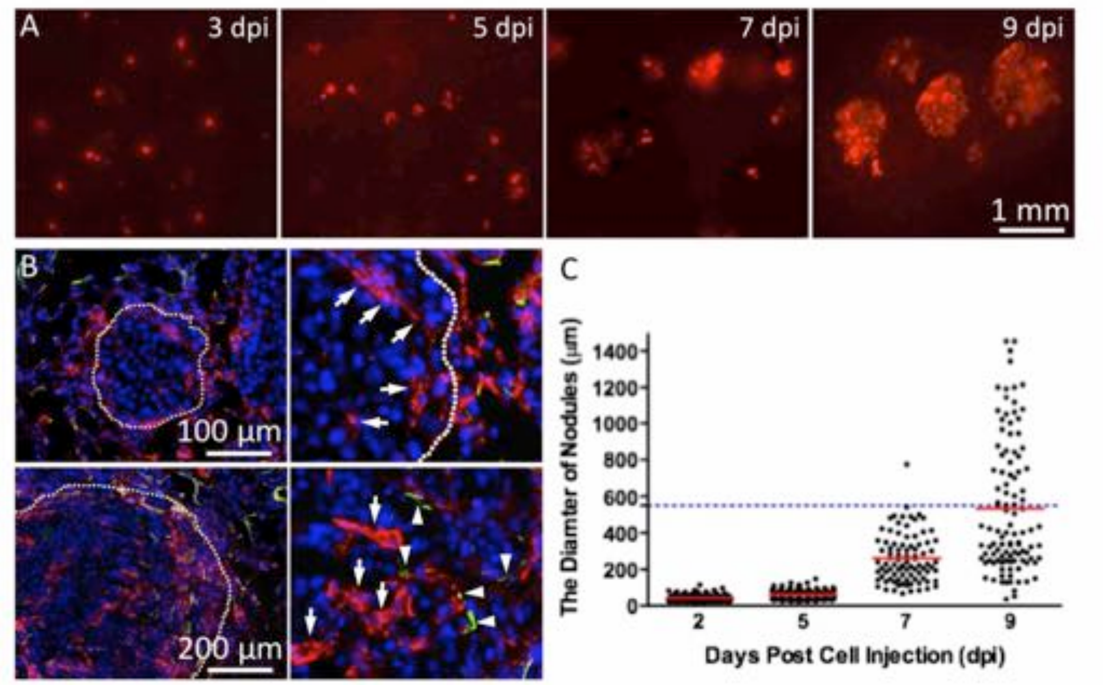

Figure S9. Tracking of the growth and blood perfusion of murine melanoma metastases in mouse lung in time. (A) Red B16 metastases on the surface of mouse lung on day 3, 5, 7 and 9 post cell implantation. (B) Blood-perfusion vessels (CD31+Lectin+) are rarely observed in metastases <500 μm in diameter, but commonly emerged in metastases > 800 μm (diameter), solid endothelial cords (arrows), blood-perfused vessels (arrowheads). T，Tumor tissue. L, Lung tissue. (C) The diameters of metastases at different time point in the mouse lung were measured and analyzed in scatter dot plot, red lines show mean diameter, blue dotted line indicates the diameter of the metastases that had its first blood-perfused vessel (Dia=553±89μm, totally n=435 metastases were checked).

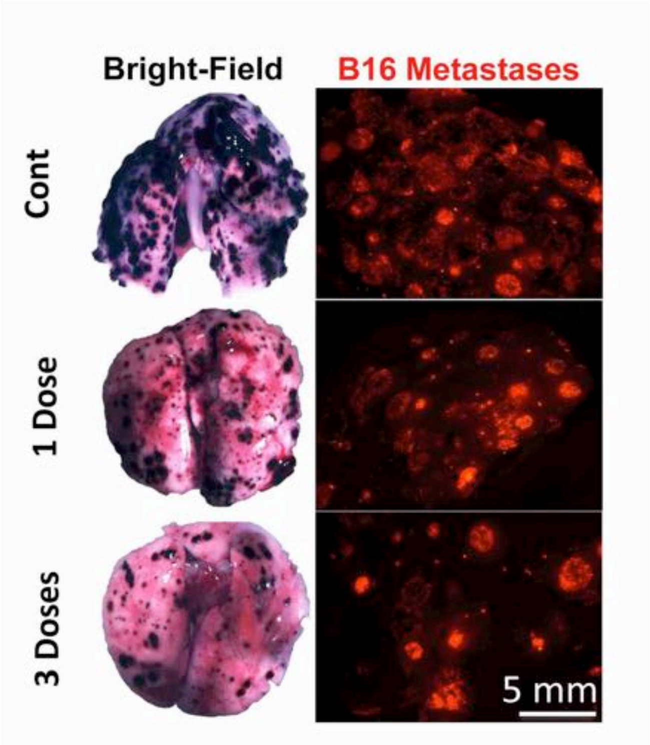

Figure S10. Stereomicroscopy showing the lungs of C57BL/6 mouse on 15 dpi. 1 dose (on 6dpi) or 3 doses (on 5, 6, 7 dpi) of SU5416 (25 mg/kg) treatment impaired the growth of B16 mouse melanoma metastases on the mouse lung on 15dpi. Vehicle treatment was set as control.

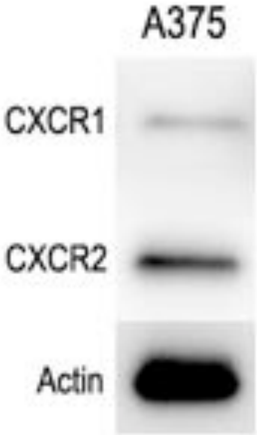

Figure S11. Expression of IL-8 receptors CXCR1 and CXCR2 on human A375 melanoma cells.

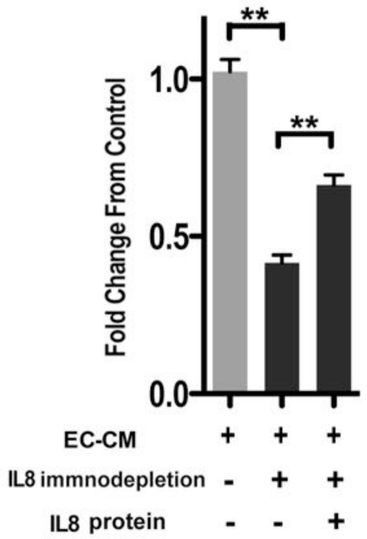

Figure S12. Effect of IL-8-immunodepleted EC-CM and the add-back of exogenous IL-8 to human A375 melanoma cell growth. **p < 0.01.

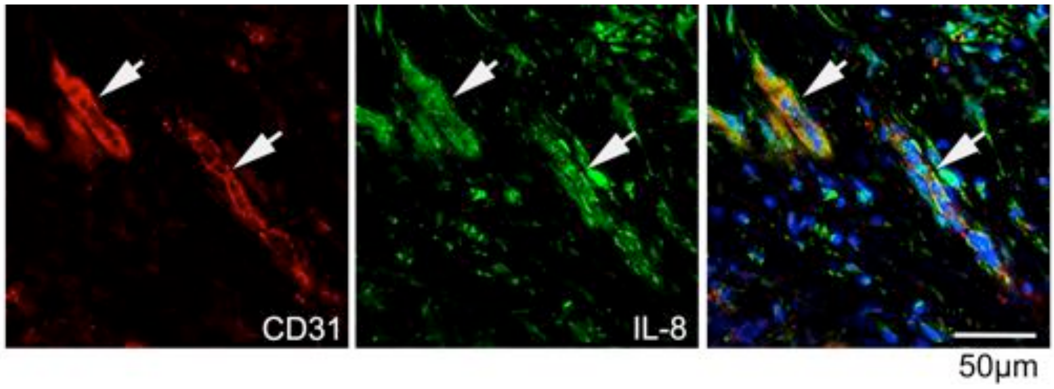

Figure S13. Expression of CD31 (red) and IL-8 (green) in human melanoma. Arrows indicate endothelial cells co-expressing CD31 and IL-8.

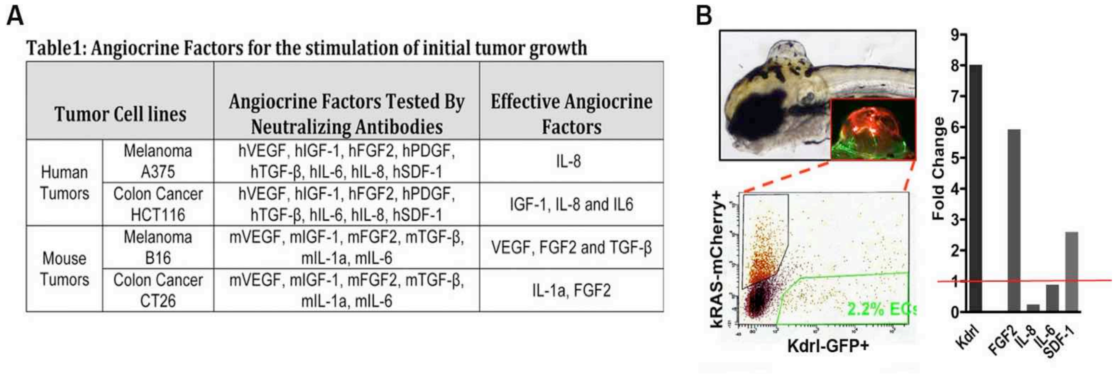

A

Table1: Angiocrine Factors for the stimulation of initial tumor growth

Tumor Cell lines		Angiocrine Factors Tested By Neutralizing Antibodies	Effective Angiocrine Factors
Human Tumors	Melanoma A375	hVEGF, hIGF-1, hFGF2, hPDGF, hTGF-β, hIL-6, hIL-8, hSDF-1	IL-8
	Colon Cancer HCT116	hVEGF, hIGF-1, hFGF2, hPDGF, hTGF-β, hIL-6, hIL-8, hSDF-1	IGF-1, IL-8 and IL6
Mouse Tumors	Melanoma B16	mVEGF, mIGF-1, mFGF2, mTGF-β, mIL-1a, mIL-6	VEGF, FGF2 and TGF-β
	Colon Cancer CT26	mVEGF, mIGF-1, mFGF2, mTGF-β, mIL-1a, mIL-6	IL-1a, FGF2

Figure S14. (A) Table 1 summarizes the effective angiocrine factors in the EC-CM identified using neutralizing antibodies. (B) The strategy for isolating the endothelial cells in endogenous zebrafish Glioma microtumors. (L) qPCR quantification of FGF2 and SDF-1 overexpression in the microtumor ECs, using GFP negative cells of the microtumors as control (red line indicates control expression level). (*$p < 0.05$, **$p < 0.01$).

Table S1. Sequences of primers used in qPCR.

Name		Sequence (5' -> 3')	Product Length
FGF2	Forward	*TACCAACCGTTTCCTTGCCA*	
	Reverse	*CGGGATACTTGCGGGATCTG*	131
IL-8	Forward	*GGCAAAATGACCAGCAAAATCATTTC*	
	Reverse	*GGTGATCCGGGCATTCATGG*	316
IL-6	Forward	*TCAACTTCTCCAGCGTGATG*	
	Reverse	*TCTTTCCCTCTTTTCCTCCTG*	73
SDF-1a	Forward	*CGCCATTCATGCACCGATTTC*	
	Reverse	*GGTGGGCTGTCAGATTTCCTTGTC*	272
β-actin	Forward	*CTTCTTGGGTATGGAATCTTGC*	
	Reverse	*GTACCACCAGACAATACAGTG*	113
kdrl	Forward	*TGTGGTCCCCTGTAGAACAAC*	
	Reverse	*GCGTTATCCACCCTGGTCAA*	180

Supplemental Experimental Procedures

Cell and Cell Culture. Mouse and human tumor cells were cultured at 37℃ in 5% CO_2 in RPMI-1640 or DMEM medium supplemented with 10% fetal bovine serum (1). Primary human endothelial cells (phECs) were isolated from the human umbilical cord vein by digestion with 0.1% collagenase as previously described (2) and were incubated in M199 medium (20% heat inactivated fetal calf serum, 100 UI ⁄mL penicillin, 100 mg⁄mL streptomycin, and 2 mM L-glutamin, 10 ng ⁄mL bFGF) in a flask coated with 1% gelatin. Primary mouse pulmonary ECs (pmECs) were isolated from 4-6-week-old wild-type C57BL/6 mice lungs by digestion with collagenase A (1.0 mg/ml) and two rounds of magnetic bead sorting (using Dynabeads coupled to anti-CD31 antibody and the Dynal MRC-L Magnetic Particle Concentrator) (3). pmEC were cultured in M199 complete, gelatin-coated flasks, and subsequently split 1:2 at each passage.

Mouse pulmonary metastatic model. A suspension of 2.5×10^5 red fluorescently labeled B16-red cells or CT26 cells in 0.1 ml of cell culture medium was injected into mouse through the tail vein. Mice were sacrificed at different time points according to the expeirments. Lungs were excised and treated if needed. The surfaces of intact lungs were examined by fluorescence Zeiss SteREO Discovery microscope (Carl Zeiss Microimaging Inc., Germany). For lungs that would be sectioned and processed for immunofluorescence and quantitative analysis, a 30% sucrose and OCT mixture (1:1; 300 μl per lung) was perfused into the mouse lung through the tracheae and then flash frozen in liquid nitrogen to fix the structure of lung pulmonary capillaries and the micrometastases.

Xenograft Model in Zebrafish. Tg(flk1: EGFP) zebrafish were bred and maintained normally (temperature, 28°C; pH 7.2–7.4; 14 hr on and 10 hr off light cycle). Wild type or red fluorescence-labeled cells were harvested at a concentration of 1×10^8 cells/ml. This mixture was loaded into a borosilicate glass needle pulled by a Flaming/Brown micropipette puller (Narishige, Japan, PN-30). 5~10 nanoliters suspension containing about 50–100 mouse tumor cells or 500 human A375 melanoma cells were implanted into each zebrafish embryo through the perivitelline

space in a single injection by using an electronically regulated air-pressure microinjector (Harvard Apparatus, NY, PL1-90). After injection, zebrafish were washed once with fish water and examined for the presence of tumor cells. For each implantation, about 50 fish were selected and transferred to 6-well plate containing 2 ml of fresh fish water and subsequently documented photographically. Fish water was changed daily, and larva that more than 5 days old were fed twice a day with grinded brine shrimp and maintained under normal fish husbandry conditions.

Imaging and neovessel quantification. Living zebrafish embryos were anesthetized by 0.003% tricaine and embedded in a dorsal, ventral, or lateral orientation in 3% methylcellulose. Digital micrographs were taken with a Zeiss Imager.Z1 fluorescence microscope (Carl Zeiss Microimaging Inc., Germany) equipped with an AxioCam MRc5 digital CCD camera (Carl Zeiss Microimaging Inc., Germany) or a Zeiss LSM 510 Meta Confocal Microscope (Carl Zeiss Microimaging Inc., Germany). Whole animal images were taken with a Zeiss Stemi 2000-C stereomicroscope with the AxioCam MRc5 digital CCD camera (Carl Zeiss Microimaging Inc., Germany). All images were taken in the same focal plane in brightfield and transmitted light passing through RFP or GFP filters. Excitation was 488 nm for GFP, 561 nm for DsRed. To clearly imaging all vessels within xenograft models, 0.5–2 μm step z-stacks (512 × 512 focal planes, 50–200 μm in depth) were acquired by using 10×(ZEISS, Plan-Neofluar) or 20×(ZEISS, Plan-Neofluar) objectives. Image capture, processing and adjustment were performed with ZEISS Axiovision rel.4.8 software. The tumor size, vessel length and vessel diameter were quantified by Axiovision Rel 4.8 software (Carl Zeiss Microimaging Inc., Germany). To evaluate the quantitative change of neovessels in microtumors, for those in mouse lung, the number of neovessels was estimated on CD31 immunostained sections; and for those implanted on the transgenic zebrafish, the number of neovessels was estimated by counting the endothelial branch points. The number of microvessels was counted under microscopy (> 20 selected areas in each group were counted) and the mean density was calculated to the average number of neovessels.

Pharmacological Treatment of Fish or Mice with SU5416. VEGFR2 specific kinase inhibitor (SU5416) (Sigma–Aldrich, Munich, Germany) was dissolved in

Dimethyl sulphoxide (DMSO: Sigma Chemical Co.). To inhibit the angiogenic sprouts in zebrafish xenografted tumors, DMSO dissolved SU5416 was directly added into the fish water at a final concentration of 2μM on the second day after tumor cell injection, DMSO alone was used as a control . Animals (>50 fish for each group) were maintained in 2 ml fish water that was changed daily. Experiments were terminated on day 5 after cell injection. For specific inhibition of the angiogenic sprouts in the micrometastases in the mouse lung, DMSO dissolved SU5416 (25mg/kg, 100 μl) was intraperitoneally injected to each mouse on day 6 (1 dose) or day 5, 6 and 7 (three doses), DMSO injection alone was set as control. Animals were sacrificed and examined on day 10 or day 15 (5 mice for each group and each time point).

EdU Assay. EdU detection for the proliferating cells in the zebrafish xenografted tumor was done using Click-iT®EdU Alexa Fluor® 488 Imaging Kit (Invitrogen, C10337). The experiments were done following the manufacturer's instructions with some modification. Briefly, Instead of using 3.7% formaldehyde in PBS to fix the samples, 1% formaldehyde in PBS was used to fix the samples at 4 °C overnight in order to protect the fluorescence of flk:mCherry. And higher concentration of EdU (100 μM) was added into the tumor cell mixtures before transplantation.

Transwell Coculture experiments. For the coculture experiments, 10,000 tumor cells were seeded into the 24-well plates, and 10,000 corresponding tumor cells or primary endothelial cells were seeded into upper compartments (transwell inserts, pore size: 0.4μm, sigma-Corning), serum reduced DMEM (2% FBS) was added into the base wells (600 μL) and the upper transwell inserts (200 μL). After 96 h of co-culture, the medium was aspirated and the cells in upper transwell inserts were fixed in 2% Formaldehyde then stained with hematoxylin, while the tumor cells in the base wells were made into single cell suspensions for counting, Ki-67 immunofluorescent staining as described below.

TCM (tumor cell conditioned medium) was generated from pure non-confluent tumor cells. EC-CM (endothelial cells conditioned medium) was generated from non-confluent HUVECs or primary mouse pulmonary ECs co-cultured with corresponding tumor cells in an upper chamber. After 48 hours of culture in DMEM (Invitrogen)

supplemented with 5% FBS, penicillin (100 U/ml), and streptomycin (100 μg/ml), Cells and debris were removed by centrifugation (5 min, 500×g), and conditional medium was then mixed with fresh DMEM medium (1:1) and was stored at −80°C.

Western blotting. The expression of PCNA (Proliferating Cell Nuclear Antigen) in tumor cells in coculture systems, VEGF expression level of tumor cells treated by VEGF siRNA or control siRNA and the detection of VEGFR2 expression both in endothelial cells and tumor cells were tested by Western blotting following the regular Western blotting procedure. The used primary antibodies are rabbit anti-PCNA (Abcam，ab15497), rabbit anti-VEGF (Abcam，ab46154), rabbit anti-VEGFR2(Santa Cruz Biotechnology, sc-505).

Quantitative PCR analysis. The expression of some of the angiocrine factors by endothelial cells in zebrafish spontaneous glioma was analyzed by q-PCR according to standard procedure and complementary DNA was generated with the Super Script First Strand Synthesis System for real-time (RT)-PCR (Invitrogen) and analyzed by quantitative RT-PCR (qRT-PCR) using SYBR® Green Real-Time PCR Master Mix (Life Technologies) and the Mx3005P QPCR System (Applied Biosystems). The GAPDH gene was used to normalize data and relative expression was calculated using the ΔΔCT method. Primer sequences are listed in Supplementary Table S1.

Immunofluorescence. Frozen tissue was cut into 8-μm-thick cross-sections, and then fixed in 100% acetone at -20℃ for 15 min. We incubated the sections with primary antibodies at 4℃ overnight. The primary antibodies were rat anti-CD31 (BD PharMingen, 561814), and rabbit anti-Ki67 (ABCAM, ab15580). Host specific Alexa Fluor 488– and 594–conjugated secondary antibodies (Invitrogen) were used to reveal the primary antibodies. The nuclei were counterstained with 4'-6-Diamidino-2-phenylindole (DAPI, Sigma). Fluorescent staining was analyzed using a Zeiss Imager.Z1 fluorescence microscope (Carl Zeiss Microimaging Inc., Germany) or Leica TCS SP5 II Confocal Microscope (Leica microsystems, Germany).

Transient siRNA Transfection. The siRNA kits were purchased from Qiagen Inc. (Valencia, CA). VEGF, sense: 5′-AUGUGAAUGCAGACCAAAGAA-TT; IL-8,

sense: 5'-ACCACCGGAAGGAACCAUC-TT; CONT, 5′-GAUAGCAAUGACGAAUGCGUA-TT. In vitro transfections were performed using the LipofectamineRNAiMAX Transfection Reagent (Life Technologies) according to the manufacturer’s protocol.

Histology Analysis. To examine the microtumors xenografted on zebrafish embryos, the xenografted zebrafish was embedded in tissue freezing medium (OCT). Frozen zebrafish were sectioned to 8μm thickness, and hematoxylin staining was performed as standard protocol. To evaluate the murine melanoma B16 metastases in the mouse lung, 5 μm paraffin sections were mounted on Superfrost Plus glass slides (Fisher Scientific) and stained with H&E as standard protocol.

Cytokine Neutralizing Assay. To neutralize cytokines in HUVEC (phECs) conditioned media, antibodies against human TGFβ (Abcam, ab27969), FGF2 (Millipore, 05-117), IL8 (Abcam, ab10769), IL6 (Abcam, ab6672), IGF1 (Abcam, ab9572), VEGFa (Millipore, 07-1420), PDGFβ (Abcam, ab9704) and SDF1 (Abcam, ab10395) were used. To neutralize cytokines in pmECs conditioned media, antibodies against mouse TGFβ (Abcam, ab64715), FGF2 (Abcam, ab33103), VEGFa (R&D, AF-493-NA), IL6 (R&D, AF-406-NA), IL-1a (R&D, AB-400-NA), and IGF1 (R&D, AF791) were used. All antibodies were prepared and applied according to the manufacturer's instructions. Antibodies and control IgG were diluted in EC-CM separately and pre-incubated for 2 hours at 37°C before adding into 96-well assay plate. Neutralizing antibodies and media were changed once after 48h. After 96 hours' incubation at standard culture conditions, the proliferation ability and number of the tumor cells in the wells was then measured either by trypsinized and counted under microscope or MTT assays.

Immunodepletions

Anti-human IL-8 Ab (5 μg/ml each) (R&D Systems, Minneapolis, MN) were used to immunodeplete the conditioned media from ECs cocultured with tumor cells. ECs supernatants were pre-cleared with protein A beads (Invitrogen, Carlsbad, CA) for 30 min, followed by overnight incubation with the IL-8 Ab. Supernatants were then incubated with protein A beads (50 μl/ml, 50% slurry) for 5 hours, followed by quick centrifugation to remove the Ab-bound beads. All incubations were done at 4°C,

rocking. Immunodepleted supernatants were then sterilized with 0.22μm filters (Millipore, Billerica, MA) for later use.

Reference

1. Yang HS, Zhang DM, Deng HX, Peng F, & Wei YQ (2010) Antitumor and anti-angiogenesis immunity induced by CR-SEREX-identified Xenopus RHAMM. *Cancer Sci* 101(4):862-868.
2. Wei YQ*, et al.* (2000) Immunotherapy of tumors with xenogeneic endothelial cells as a vaccine. *Nat Med* 6(10):1160-1166.
3. Sobczak M, Dargatz J, & Chrzanowska-Wodnicka M (2010) Isolation and culture of pulmonary endothelial cells from neonatal mice. *J Vis Exp* (46).
